# Acaricide Resistance Development in *Rhipicephalus (Boophilus) microplus* (Acari: Ixodidae) Populations against Amitraz and Deltamethrin on Communal Farms of the King Sabata Dalindyebo Municipality, South Africa

**DOI:** 10.3390/pathogens12070875

**Published:** 2023-06-26

**Authors:** William Diymba Dzemo, Patrick Vudriko, Tsepo Ramatla, Oriel Thekisoe

**Affiliations:** 1 Department of Biological and Environmental Sciences, Faculty of Natural Sciences, Walter Sisulu University, Mthatha 5117, South Africa; 2 Unit for Environmental Sciences and Management, North-West University, Potchefstroom 2531, South Africa; ra21205450@gmail.com (T.R.); oriel.thekisoe@nwu.ac.za (O.T.); 3 Research Center for Tropical Diseases and Vector Control, Department of Veterinary Pharmacy, Clinics and Comparative Medicine, College of Veterinary Medicine, Animal Resources and Biosecurity, Makerere University, Kampala P.O. Box 7062, Uganda; patrick.vudriko@mak.ac.za

**Keywords:** cattle tick, resistance development, communal farming, acaricide, *Rhipicephalus microplus*, amitraz, deltamethrin

## Abstract

Chemical acaricides are widely used to control ticks and tick-borne pathogens in cattle. However, prolonged and indiscriminate use of these chemicals inevitably leads to the selection of resistant ticks. In-vitro bioassays (adult and larval immersion tests) were conducted to assess amitraz and deltamethrin resistance in *Rhipicephalus (Boophilus) microplus* populations from communal farms of the King Sabata Dalindyebo municipality of South Africa. Data generated on percentage inhibition of oviposition (%IO) revealed that all the tick populations assessed showed resistance (%IO ≤ 95%) to at least one of the acaricides. All six tick populations assessed for efficacy (%IO ≥ 95%) at the DD) with deltamethrin were resistant (%IO ≤ 95%) and only one of the six tick populations assessed for efficacy with amitraz was susceptible. Based on the resistance ratios (RR), the adult immersion test detected amitraz and deltamethrin resistance in three (RR ranging from 2.30 to 3.21) and five (RR ranging from 4.10 to 14.59) of the six tick populations, respectively. With the larval immersion test, deltamethrin and amitraz resistance (larval mortality < 90% at the DD) was detected in all four and three of four *R. (B.) microplus* populations assessed, respectively. These data are critical for the design of an effective and sustainable tick control strategy on the communal farms.

## 1. Introduction

*Rhipicephalus* (*Boophilus) microplus* is one of the most economically important ectoparasites infesting and affecting the livestock production industry in the Eastern Cape Province (ECP) of South Africa [1]. This invasive tick species has a high adaptability in humid climatic regions, where it has displaced the indigenous *R.* (*Boophilus*) *decoloratus* in most parts of the ECP [2]. Cattle infestations by *R. (B.) microplus* result in heavy production losses to farmers, both directly (anorexia, anemia, toxicosis, damaged hides, reduced milk, and meat production) and indirectly through morbidity due to pathogens such as *Babesia* spp. and *Anaplasma* spp. that cause tick-borne diseases (TBDs) [3,4]. Annual global economic losses resulting from ticks and tick-borne diseases have been estimated at USD 13–14 billion [5,6,7]. A recent study by Makwarela et al. [8] reported that in South Africa, economic losses in the livestock industry as a result of tick infestations and TBDs are estimated to exceed USD 33 million per year (ZAR 500 million).

The majority of livestock kept in the ECP of South Africa is in communal areas, where animals belonging to different owners graze on vegetation in unfenced communal land [9]. The control of cattle ticks and TBDs in these areas is mainly through the dipping of animals in chemical acaricides provided by the provincial government at communal dipping tanks [10]. Synthetic pyrethroids (SPs), organophosphates (OPs), and amitraz are the three most commonly used groups of acaricide chemicals in communal areas of South Africa [9]. Continuous and indiscriminate use of these chemicals in the control of ticks on cattle has led to the rapid build-up of resistance to a majority of acaricides [4,11]. *Riphicephalus (B.) microplus* is a single host tick, with a short life cycle and high reproductive rate, entailing frequent acaricide treatments [12]. Frequent subjection of large proportions of these tick populations to acaricides promotes selection for resistant ticks. This negatively affects tick control programs and threatens the efficacy and life span of currently available acaricide chemical products [13]. In South Africa, chemical acaricides for the control of ticks on cattle have been in use for more than a century [14], and remain to be the predominant component of tick control programs in the near future [15]. Therefore, for optimum and strategic use of acaricides, to retard the development of resistance in ticks, it is necessary to monitor acaricide resistance in the field [11]. For this reason, the Food and Agriculture Organisation (FAO) [16] has recommended two in-vitro diagnostic tests including the larval packet test (LPT) and adult immersion test (AIT) for the assessment of field efficacy of acaricides in tick populations [16]. The larval immersion test (LIT), a modification of the LPT developed by Shaw (1966) [17], has been used in South Africa for a nationwide acaricide-resistant survey [9]. The AIT [18] requires a large number of engorged female ticks and provides results within 2 to 3 weeks. The LPT [19] and LIT [17] both involve the use of larvae and require 5 to 6 weeks to complete.

The control of cattle ticks on communal farms of the ECP of South Africa is constantly being challenged by the manifestation of tick-acaricide failure [10], which may probably be an indication of acaricide resistance by these field ticks. However, there have been few published reports [9,20,21] on acaricide resistance development in field tick populations on communal farms of the ECP during the last two decades. In order to get up-to-date information, this study was designed to assess the acaricide resistance status of *R. (B.) microplus* populations from selected communal farms of the King Sabata Dalindyebo Municipality (KSDM) of ECP, South Africa.

## 2. Materials and Methods

### 2.1. Study Area

**Figure 1:**
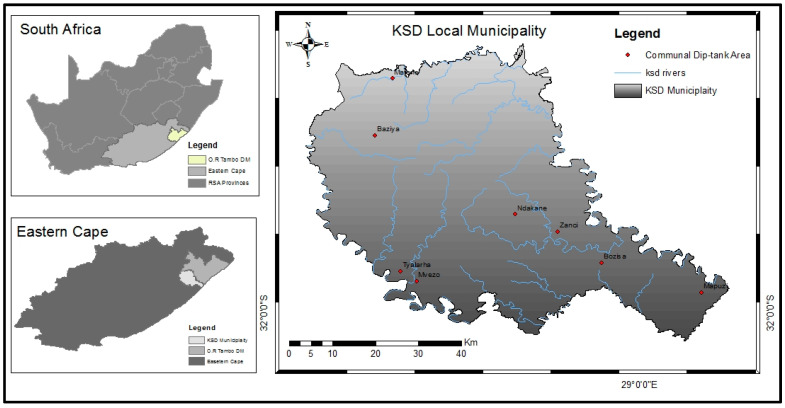
Communal farms/dip tank areas from which ticks were collected.

The KSDM is located in the Oliver Reginald Tambo (OR Tambo) District of the ECP, South Africa (31.54639°; 28.67528°) (Figure 1). Its topography varies with hills and mountains beyond the Indian Ocean coastline, averaging 764 m of altitude. Climatic conditions vary with distance and elevation away from the Indian Ocean. The coastal areas have a tropical climate while the inland areas are temperate. Annual rainfall generally exceeds 800 mm in coastal areas and decreases as one moves inland. The temperature fluctuates from an average of 14.3 °C to 25.3 °C in January and 1.8 °C to 21.4 °C in July [22]. The vegetation is characterized by upland and coastal grassland, with sporadic forests. The main agricultural activity is livestock (cattle, sheep, and goats) rearing in communal areas. The study area has an estimated cattle population of about 98,000, consisting mainly of Nguni (local indigenous breed) and non-descript cattle breeds such as Bonsmara [23]. Dipping and inspection of cattle for ticks are done 2 to 4 and 1 to 2 times per month during the summer in the coastal and inland communal farming areas, respectively [24]. 

### 2.2. Collection and Handling of Rhipicephalus (Boophilus) spp. Field Populations

The study sample consisted of 8 farms (4 each from inland and coastal areas) that were randomly sampled from a total of 40 with reported cases of high tick-acaricide failure and TBDs (Figure 1). Information on communal farms with reported cases of high tick-acaricide failure and TBDs was obtained from the OR Tambo District veterinary authorities. Prior to tick collection, physical contact was established with animal health technicians (AHTs) or community animal health workers (CAHWs) in charge of the dipping and inspection of cattle at the selected farms or dipping tanks. Through the AHTs and CAHWs, verbal consent was obtained from cattle owners and dates for tick collection were scheduled. Animals brought for dipping by each farmer were restrained in a cattle crush prior to tick collection. All visible fully engorged adult female *Rhipicephalus (B.)* spp. with a length size that was greater than 4 mm were obtained from animals that were not agitated and restless. A few male *Rhipicephalus (B.)* spp. were also collected and were used mainly for the morphological identification of tick species. The collection of ticks was conducted during the peak tick activity (between the months of February to April 2019) and early in the morning (5–7 am) before cattle were dipped and released to graze. Ticks collected were placed in perforated plastic containers to allow for air and moisture movement. The date of collection and dip tank name were affixed on the perforated containers and transported to the laboratory inside a cool box with ice blocks wrapped in paper towels [16]. In the laboratory, ticks were placed on a sieve and washed with tap water to remove feces and any eggs that might have been laid during transportation. Ticks were allowed to dry by placing them on paper towels.

### 2.3. Identification of Tick Species

#### 2.3.1. Morphological Identification of the Tick Species

The male and engorged female ticks were morphologically identified with a dissecting microscope (Olympus^®^ SZX10, Tokyo, Japan) using keys and descriptions developed by Walker et al. [25]. Hypostomal dentition (ventral and central structure of the mouthparts) and adanal spur were the key morphological features for the identification of *Rhiphicephalus (Boophilus)* spp. [25]. In *R.* (*B*.) *decoloratus*, the hypostomal teeth dentition is arranged in 3 + 3 columns while they are in 4 + 4 columns in *R*. (*B*.) *microplus* (Supplementary Figure S1). The *R*. (*B*.) *microplus* has no protuberance bearing setae on the internal margin of palp article 1, while this protuberance is present and bearing setae in *R*. (*B*.) *decoloratus* (Supplementary Figure S1).

#### 2.3.2. Molecular Identification of the Tick Species

For each field tick population, genomic DNA was extracted from two of the morphologically identified specimens of *R. (B.) microplus* adults that had been stored in 70% *v*/*v* ethanol, using the modified salting-out method [26]. The concentration and quality of the 16 extracted DNA samples were determined using the NanoDrop One spectrophotometer (Thermo Fischer Scientific Inc., USA) and stored at −35 °C.

Polymerase chain reaction (PCR) was used for the identification of the tick species targeting the *Cytochrome c Oxidase subunit 1* (*CO1*) and *Internal Transcribed Spacer 2* (*ITS2*) gene fragments [27,28]. For the amplification of the 710 bp (bp) segment of the *CO1* gene, LCO1490 (5′-GGTCAACAAATCATAAAGATATTGG-3′) and HCO2198 (5′-TAAACTTCAGGGTGACCAAAAAATCA-3′) were used as the forward and reverse primers, respectively [27]. To amplify the 900–1200 bp segment of the *ITS2* gene, *ITS2* F 5′-TGTGAACTGCAGGACACATGAA-3′ (forward) and ITS2R 5′-ATGCTTAAATTTAGGGGGTAGTC-3′ (reverse) primers were used [28].

The PCR was performed in a 25 μL reaction mixture containing 12.5 μL of Amplitaq Gold^®^ 360 Master Mix (Applied Biosytems, Waltham, MA, USA), 2 μL primer mix with a concentration of 10 μM for each primer, 8.5 μL of nuclease-free water, and 2 μL of the template DNA. The PCR thermal cycling conditions included an initial denaturation at 95 °C for 10 min and 35 cycles of denaturation at 95 °C for 30 s, annealing was at 47 °C for 30 s for the *CO1* gene and 58 °C for 30 s for the *ITS2* gene, and extension at 72 °C for 1 min. The elongation was further extended for 7 min at 72 °C followed by a 4 °C hold.

The PCR amplicons were visualized using electrophoresis on 1% *w*/*v* agarose gel that was stained with ethidium bromide in TAE buffer (40 mM Tris-acetat, 1 mM EDTA, pH 8.0) and viewed under UV light. Four randomly selected PCR-positive aliquots (two for *CO1* and *ITS2* genes) were sequenced in both directions at Inqaba Biotech Industries (Pretoria, South Africa). 

### 2.4. Rearing of Engorged Female Ticks for Eggs and Larvae 

Ten fully engorged female *R. (B.) microplus* from each sample farm were each placed in three separate 100 mL conical flasks that were firmly closed with cotton wool plugs to allow for airflow and prevent the escape of emerged larvae after egg hatching. The flasks were placed in an incubator and maintained at 27 to 28 °C and 85 to 95% relative humidity for egg laying. After 7 days of incubation, the engorged female ticks were removed and placed into new conical flasks. The flasks containing each week’s egg production were labeled with the date. This was done to enable larval age uniformity for the larval immersion test. For egg hatching, the flasks containing the eggs were kept under similar conditions of incubation for 21–28 days [16]. The date of hatching was considered to be the day when an estimated 75% of the eggs hatched. The percentage hatching rate in each flask was determined by observing the proportion of larvae in relation to eggs not hatched [18]. Live larvae of 14–21 days of age were used for assessment of acaricide resistance [16].

### 2.5. Acaricides Used in the Study 

According to information from the OR Tambo District veterinary authorities and Dzemo et al. [24], acaricide chemical compounds such as Deca-tix^®^3 (Deltamethrin 2.5% *m*/*v*), Delete-X5^®^ (Deltamethrin 5% *m*/*v*), and Taktic^®^ (Amitraz 12.5% *m*/*v*) have been extensively used at the communal dipping tanks for the treatment of ticks on cattle. Commercially available preparations of 5% *m*/*v* deltamethrin (Delete^®^-X5-Intervet South Africa (Pty) Ltd., Kempton Park, South Africa) and 12.5% *m*/*v* amitraz (Triatix^®^-Coopers Veterinary Products (Pty) Ltd., Kempton Park, South Africa) were used in the study. Delete^®^-X5 was obtained from batches of acaricides procured by the Provincial government directly from suppliers. Triatix^®^ was purchased from registered local veterinary drug shops. Acaricide chemicals procured by the Provincial government and those sold at veterinary drug shops have been screened for quality (concentration of active ingredient) by the South African National Pesticide Registration Authority, Registrar Act 36 of 1947. Only batches that are compliant with label claims are registered and supplied to users for the control of pests. The registration numbers according to Act 36 of 1947 for Delete^®^-X5 and Triatix^®^ are G3279 and G3189, respectively.

Diagnostic doses (DD) used in the study were the manufacturer’s recommended concentration of 0.025 (250 ppm) and 0.005% (50 ppm) *m*/*v* for amitraz and deltamethrin, respectively. A two-fold serial dilution, consisting of seven concentrations (3 above DD, 3 below DD, and the recommended manufacturer’s DD concentration) of the acaricides, was prepared in distilled water from a 1% *m*/*v* stock solution. The different acaricide concentrations assessed were 2000, 1000, 500, 250, 125, 62.25, and 31.25 ppm for amitraz and 400, 200, 100, 50, 25, 12.5, and 6.25 ppm for deltamethrin. 

### 2.6. Adult Immersion Test (AIT) 

The AIT was conducted as described by Drummond et al. [18] with minor modifications to assess acaricide resistance in engorged females of *R. (B.) microplus.* Commercial formulations of acaricides were diluted in distilled water, in the same way as the stock solution was diluted [18], instead of 25%, 65% xylene, and 10% Triton-X100 [16]. Another modification related to the immersion time of 30 min, similar to the modified AIT in FAO [16], instead of 30 s of the classical Drummond AIT as described by Drummond et al. [18]. 

Engorged female ticks were randomly assigned to form as many groups of tens, as the acaricide concentrations were to be assessed in triplicates. Each group of 10 ticks was weighed using a digital laboratory scale (Mettler PJ3600 DeltaRange^TM.^, Washington, DC, USA). The groups of 10 ticks were immersed in 10 mL of the different acaricide concentrations and distilled water (negative control) for 30 min in a 50 mL beaker and gently agitated. Afterward, the acaricide solutions were drained, and the recovered ticks were dried on paper towels. The dried/treated ticks were then pasted on double-sided tape inside plastic Petri dishes, with their dorsal surfaces facing downwards. Petri dishes with the treated ticks were closed with perforated lids, labeled accordingly, and stored for 20 days [16] in an incubator maintained at 27 to 28 °C and 85 to 95% relative humidity for oviposition. Ticks that did not lay eggs after 20 days of incubation were considered dead [16]. This was confirmed using physical observation under the microscope for lack of movement of limbs despite stimulation from light. The following parameters were recorded and compared:

(a) The number of live females and percentage of adult tick mortality per replicate.
(b) The egg mass (mg) laid by treated ticks per replicate.
(c) Index of fertility (IF)—a measure of egg-laying capacity of the treated ticks expressed as the weight of eggs laid (mg)/weight of female ticks (mg).
(d) Percentage inhibition of oviposition (%IO) = [(IF control group − IF treated group)/(IF control group) × 100].

Acaricide concentrations were considered efficient when efficacy (percentage of inhibition of oviposition) was higher than or equal to 95% [18]. 

Due to the unavailability of sufficient fully engorged female *R. (B.) microplus* ticks (size > 4 mm), and for each acaricide compound, the AIT was conducted on six different field populations from the eight initially sampled farms.

### 2.7. Larval Immersion Test (LIT) 

The LIT was conducted as originally described by Shaw [17], where treated tick larvae were incubated for 17–18 hrs before assessing for mortality. However, in this study, modifications were made with regard to the incubation period of 24 hrs for deltamethrin, and 48 hrs for amitraz [16], to allow for sufficient exposure time. 

Using a soft paintbrush, approximately 100 larvae 14 to 21 days old were gently transferred from a conical flask that was holding them onto a filter paper and placed inside a Petri dish. An acaricide dilution or distilled water (control) was gently agitated and 10 mL of it was withdrawn with a syringe. An amount of 5 mL of the 10 mL acaricide dilution or distilled water was sprinkled onto the larvae on the filter paper. A second filter paper was placed over the larvae and sprinkled with the remaining 5 mL of acaricide dilution. The filter paper–larvae–filter paper sandwich was set aside for an immersion period of 10 min (the test procedure was replicated twice with the same acaricide dilution). When the immersion period was over, the filter paper sandwich was removed from the Petri dish, opened, and both filter papers were placed on dry paper towels for moisture absorption. After the air drying, all the treated larvae were carefully transferred by sweeping with the fine brush into a Whatman filter paper envelope closed on the sides with metal clips. The filter paper envelope with the treated tick larvae was sealed with metal clips and labeled accordingly. Envelopes containing treated tick larvae were packed in sequence inside a small cardboard box of 10 cm (L) × 5 cm (W) × 4 cm (H) partition in such a way that tick larvae treated with the different dilutions did not make contact with each other. The cardboard box containing the envelopes was placed inside an incubator maintained at 27 to 28 °C and 85 to 95% relative humidity [16]. The negative control packets were stored in a separate incubator. After 24 h (deltamethrin) and 48 h (amitraz) of incubation, an assessment of percent larval mortality was conducted by counting the dead and live larvae. Larvae that were unable to walk after stimulation with light on the surface of the filter paper were considered dead. For the assessment of resistance against deltamethrin and amitraz, a total of 24 tests were performed (triplicates for each acaricide dilution). Abbott’s formula was applied when the percentage of larvae mortality in the control (distilled water) was between 5 and 10% [16] as follows:

Corrected percent mortality = [(% test mortality − % control mortality)/(100 − % control mortality) × 100].

When the percentage of larval mortality in the control was greater than 10%, the entire results were discarded. Field tick populations were considered to be susceptible if the percentage of larval mortality was greater than 90% [29].

### 2.8. Data Analysis

For genetic identification of the ticks, nucleotide sequences were extracted and converted from AB1 format to FASTA format using Molecular Evolutionary Genetics Analysis Version 7 (MEGA7). Mixed bases were edited to their appropriate base pairs [30]. The resulting sequences were subjected to the nucleotide Basic Local Alignment Search Tool (BLASTn) to align against references in the GenBank of National Center for Biotechnology Information (NCBI) (http://www.ncbi.nlm.nih.gov/BLASTn, accessed on 28 April 2019) for high-similarity sequences confirming the identity of the ticks.

Data from the AIT and LIT were introduced into GraphPad Prism 8 for Windows version 8.01 statistical software for analyses. The significance of the mean mass of engorged female ticks, IF, and %IO between groups were determined by one-way ANOVA at the 5% level of significance. Multiple comparisons between group means were conducted with the Tukey test. The resulting data were presented as mean ± SE for the variables assessed. The percentage of adult and larval mortality data were submitted to Probit analysis. Regression analysis was applied to the normalized transformed percent mortality vs logarithm concentration data to determine the LC_50%_ values with their respective 95% confidence intervals (95% CI), the slope of regression line, and coefficients of determination (R^2^). The LC_50%_ value of the reference-susceptible *R. (B.) microplus* IVRI-I line [6,31] was used to determine resistance ratios (RRs) for deltamethrin and amitraz with the AIT. The RRs were calculated with the following formula described by Kumar et al. [31]:

Resistance ratio (RR) = LC_50%_ value of field ticks/LC_50%_ value of reference susceptible ticks.

To provide an estimate of the relative level of resistance in each field tick population, the resistance ratio was designated as significant when the 95% confidence interval of the LC_50%_ value of the reference strain and field tick population did not overlap [32]. 

## 3. Results

### 3.1. Morphological and Molecular Identification of Tick Species 

As many as 960 to 1084 fully engorged *R*. (*B*.) *microplus* were obtained from cattle at five of the eight farms sampled. On the other hand, about 280 to 310 ticks were harvested from the remaining three farms, due to the unavailability of a sufficient number of suitable (size > 4 mm) fully engorged female ticks. 

The *R*. (*B*.) *microplus* CO1 gene representative sequences (Bozisa and Mapuzi) were 99.12 to 99.85 % identical to that of *R*. (*B*.) *microplus* (accession no. KY678117.1) from South Africa. The *R*. (*B*.) *microplus* ITS2 gene representative sequences (Zanci and Mpafane) had a 98.17 % similarity with the Chinese and South African *R*. (*B*.) *microplus* (accession no. MK224566.1, MK224560.1, MG721035.1, and KY457506.1). BLASTn results of the top three matches from the query sequences obtained from the amplification of CO1 and ITS2 genes are presented in Table 1.

### 3.2. Amitraz and Deltamethrin Resistance Status from Field-Derived R. (B.) microplus Populations Using Adult Immersion Test (AIT)

The effect of immersing adult engorged females of *R*. (*B*.) *microplus* in increasing concentrations of amitraz and deltamethrin on reproductive parameters, including index of fertility (IF) and inhibition of oviposition (IO), is represented in Table 2 and Table 3, respectively. Generally, total mortality of ticks did not occur at the DD for both acaricide compounds. All eight field tick populations showed resistance (%IO ≤ 95%) to at least one of the acaricide compounds. All six field tick populations assessed for efficacy with deltamethrin were resistant (%IO ≤ 95%) at the DD and 2XDD (Table 3). Meanwhile, only one (Mpafane) of the six field tick populations assessed for efficacy with amitraz was susceptible (%IO ≥ 95%) at the DD and 2XDD (Table 2). Notably, at the 8XDD, amitraz and deltamethrin could not cause total mortality to the exposed engorged female ticks. Two and one of the field tick isolate(s) assessed for efficacy with amitraz and deltamethrin, respectively, showed statistically significant differences (*p* < 0.05) between the DD and control treatments (Table 2 and Table 3). 

Values of the concentration–mortality slopes, LC_50%_ (lethal concentration to kill 50% of the population) values with their 95% confidence intervals (CI 95%), and R^2^ (coefficients of determination) for each tick population assessed using AIT for resistance against amitraz and deltamethrin are shown in Table 4. LC_50%_ values ranged from 46 to 531 ppm for amitraz and 11 to 196 ppm for deltamethrin. The resistance ratio (RR) values of three and five of the six *R*. (*B*.) *microplus* field populations assessed for amitraz and deltamethrin resistance, respectively, were designated as significant, because the 95% confidence interval of the LC_50%_ value of the reference strain and field tick population did not overlap (Table 4). A majority of these resistant field tick populations emanated from the coastal farms where cattle were chemically treated for ticks on a weekly or fortnight basis during the summer season. The coefficients of determination values of all the estimations were ≥70%, indicating good fitting of the data in the Probit model. 

**Table 1:** BLASTn results of top three matches of the query sequences from *R. (B.) microplus* populations.

Communal Farm	Target Gene	Morpological Identifiction	Blastn Description (Country)	Maximum Score	Total Score	Query Cover (%)	E-Value	Percent Identity (%)	Accession No.
Bozisa	*CO1*	*R. (B.) microplus*	*Rhipicephalus microplus* (South Africa)	1221	1221	99	0.0	99.12	KY678117.1
			*Rhipicephalus microplus* (Brazil)	1221	1221	99	0.0	99.12	KC503261.1
			*Rhipicephalus microplus* (Kenya)	1221	1221	99	0.0	99.12	MT430986.1
Mapuzi	*CO1*	*R. (B.) microplus*	*Rhipicephalus microplus* (South Africa)	1236	98	98	0.0	99.85	KY678117.1
			*Rhipicephalus microplus* (Brazil)	1236	98	98	0.0	99.85	KC503261.1
			*Rhipicephalus microplus* (Kenya)	1236	98	98	0.0	99.85	MT430986.1
Zanci	*ITS2*	*R. (B.) microplus*	*Rhipicephalus microplus* (China)	1995	2109	98	0.0	98.17	MK224582.1
			*Rhipicephalus microplus* (China)	1995	2109	98	0.0	98.17	MK224580.1
			*Rhipicephalus microplus* (China)	1995	2109	98	0.0	98.17	MK224579.1
Mpafane	*ITS2*	*R. (B.) microplus*	*Rhipicephalus microplus* (China)	1995	2109	98	0.0	98.17	MK224579.1
			*Rhipicephalus microplus* (China)	1995	2109	98	0.0	98.17	MK224582.1
			*Rhipicephalus microplus* (China)	1995	2109	98	0.0	98.17	MK224580.1

**Table 2:** Effect of amitraz on index of fecundity (IF) and percentage inhibition of oviposition (%IO) of *R. (B.) microplus*.

Agro-Climatic Region	Communal Farm Area	Index of Fecundity (IF ± SE) ^a^								
		Amitraz Concentration (ppm)								
		2000	1000	500	250 *	125	62.5	31.25	Control	*p*-Value
**Inland Region**	Mpafane	0.0043 ± 0.0030a	0.0044 ± 0.0024a	0.0107 ± 0.0091ab	0.0145 ± 0.0024abc	0.0342 ± 0.0074abc	0.0697 ± 0.0040bc	0.0758 ± 0.0061c	0.3288 ± 0.0325d	*p* < 0.001
	Tyalarha	0.1054 ± 0.0164a	0.1428 ± 0.0213ab	0.1945 ± 0.0819abc	0.2851 ± 0.0845abc	0.3494 ± 0.0325bc	0.3842 ± 0.0249c	0.3819 ± 0.0425c	0.4084 ± 0.0189c	*p* < 0.01
	Mveso	0.0435 ± 0.0171a	0.0456 ± 0.0116a	0.0475 ± 0.0133a	0.1024 ± 0.0245ab	0.1164 ± 0.0261ab	0.1983 ± 0.0235b	0.3288 ± 0.0325c	0.3943 ± 0.0187c	*p* < 0.001
**Coastal Region**	Bozisa	0.0730 ± 0.0147a	0.1065 ± 0.0409a	0.0907 ± 0.0272a	0.0980 ± 0.0277a	0.1487 ± 0.0249ab	0.1948 ± 0.0529ab	0.2013 ± 0.0399ab	0.2737 ± 0.0404b	*p* < 0.05
	Zanci	0.0152 ± 0.0089a	0.0522 ± 0.0036a	0.0932 ± 0.0344ab	0.2172 ± 0.0084bc	0.2348 ± 0.0510c	0.2625 ± 0.0180c	0.2848 ± 0.0220c	0.3365 ± 0.0255c	*p* < 0.001
	Mapuzi	0.0000 ± 0.0000a	0.0009 ± 0.0009a	0.0331 ± 0.0031a	0.1093 ± 0.0128b	0.1128 ± 0.0129b	0.1156 ± 0.0124b	0.1363 ± 0.0094b	0.1504 ± 0.0253b	*p* < 0.001
	**Percentage Inhibition of Oviposition (%IO ± SE) ^b^**									
**Inland Region**	Mpafane	98.83 ± 0.73a	98.53 ± 0.83a	97.18 ± 2.28ab	95.54 ± 0.81ab	89.34 ± 2.83b	78.44 ± 2.25c	76.81 ± 1.42c	0.00 ± 0.00d	*p* < 0.001
	Tyalarha	74.46 ± 2.85a	64.90 ± 5.25ab	50.76 ± 11.12ab	28.50 ± 12.42ab	13.50 ± 11.77ab	4.96 ± 10.73b	2.81 ± 4.91b	0.00 ± 0.00b	*p* < 0.05
	Mveso	88.75 ± 4.39a	88.11 ± 3.57a	87.56 ± 4.07a	73.59 ± 7.23ab	57.33 ± 7.82b	50.03 ± 3.74b	16.44 ± 7.93c	0.00 ± 0.00c	*p* < 0.001
**Coastal Region**	Bozisa	72.07 ± 6.76a	63.59 ± 9.78a	62.30 ± 7.14a	59.20 ± 6.96a	42.97 ± 2.93ab	31.87 ± 10.30ab	25.81 ± 11.31ab	0.00 ± 0.00b	*p* < 0.05
	Zanci	95.75 ± 2.57a	84.47 ± 0.21a	70.67 ± 2.92ab	34.24 ± 7.72bc	31.82 ± 10.62bc	21.03 ± 7.88c	14.15 ± 5.30c	0.00 ± 0.00c	*p* < 0.001
	Mapuzi	100.0 ± 0.00a	92.20 ± 0.80a	77.49 ± 1.55a	26.14 ± 4.60b	19.16 ± 7.98b	17.01 ± 8.49b	6.51 ± 8.98b	0.00 ± 0.00b	*p* < 0.001

* Manufacturer recommended dose; ^a^ index of fecundity (IF) = mass of eggs laid/mass of engorged females. ^b^ %IO = [(IF control − IF treated)/(IF control) × 100]. SE: standard error. Mean IF/%IO followed by same letters were not statistically significant (*p* > 0.05).

**Table 3:** Effect of deltamethrin on index of fecundity (IF) and percentage inhibition of oviposition (%IO) of *R. (B.) microplus*.

Agro-Climatic Region	Communal Farm Area	Index of Fecundity (IF ± SE) ^a^								
		Deltamethrin Concentration (ppm)								
		400	200	100	50 *	25	12.5	6.25	Control	*p*-Value
**Inland Region**	Mpafane	0.0411 ± 0.0129a	0.1130 ± 0.0114a	0.1141 ± 0.0448a	0.1253 ± 0.0216a	0.1483 ± 0.0158a	0.1539 ± 0.0232a	0.1677 ± 0.0373a	0.3288 ± 0.0325b	*p* < 0.001
	Tyalarha	0.2516 ± 0.0121a	0.3156 ± 0.0152ab	0.3521 ± 0.0230bc	0.3760 ± 0.0200bc	0.4139 ± 0.0154bc	0.4174 ± 0.0244c	0.4347 ± 0.0274c	0.4506 ± 0.0201c	*p* < 0.001
	Baziya	0.0806 ± 0.0062a	0.1264 ± 0.0197ab	0.1804 ± 0.0382ab	0.2867 ± 0.0937abc	0.3058 ± 0.0109bc	0.3400 ± 0.0438bc	0.3601 ± 0.0414bc	0.3943 ± 0.0186c	*p* < 0.001
**Coastal Region**	Ndakana	0.0356 ± 0.0217a	0.1260 ± 0.0210ab	0.2039 ± 0.0214abc	0.1806 ± 0.0727abc	0.2125 ± 0.0587abc	0.2292 ± 0.0292bc	0.2392 ± 0.0218bc	0.3149 ± 0.0117c	*p* < 0.001
	Zanci	0.0756 ± 0.0400a	0.1040 ± 0.0133a	0.1179 ± 0.0215ab	0.1672 ± 0.0458ab	0.2521 ± 0.0311bc	0.2514 ± 0.0267bc	0.2609 ± 0.0206bc	0.3365 ± 0.0254c	*p* < 0.001
	Mapuzi	0.0443 ± 0.0219a	0.0889 ± 0.0338ab	0.1150 ± 0.0206ab	0.1687 ± 0.0224ab	0.1733 ± 0.0127b	0.1835 ± 0.0338b	0.1987 ± 0.0391b	0.2405 ± 0.0247b	*p* < 0.01
**Percentage Inhibition of Oviposition (%IO ± SE) ^b^**										
	Mpafane	86.83 ± 4.77a	65.61 ± 1.01a	63.51 ± 6.02a	59.98 ± 9.61a	54.59 ± 4.62a	51.52 ± 10.46a	49.30 ± 10.14a	0.00 ± 0.00b	*p* < 0.001
	Tyalarha	44.12 ± 1.72a	29.59 ± 5.22ab	21.05 ± 9.02ab	16.59 ± 1.58ab	7.53 ± 2.32b	6.65 ± 3.88b	3.15 ± 3.32b	0.00 ± 0.00b	*p* < 0.001
	Baziya	79.45 ± 1.93a	68.17 ± 4.15ab	55.00 ± 7.85abc	24.69 ± 7.83bcd	22.35 ± 1.26bcd	14.30 ± 7.65bcd	9.18 ± 7.35cd	0.00 ± 0.00d	*p* < 0.001
	Ndakana	88.19 ± 7.58a	59.66 ± 7.23ab	35.21 ± 6.35abc	43.89 ± 10.97abc	31.69 ± 9.28abc	27.45 ± 7.96bc	23.90 ± 6.93bc	0.00 ± 0.00c	*p* < 0.001
	Zanci	78.58 ± 10.15a	68.29 ± 5.78ab	64.54 ± 6.85abc	48.60 ± 6.59abc	25.65 ± 3.82bcd	23.01 ± 4.54bcd	22.46 ± 1.12cd	0.00 ± 0.00d	*p* < 0.001
	Mapuzi	80.33 ± 9.88a	59.54 ± 6.92abc	49.58 ± 10.58abc	28.07 ± 12.50abc	27.15 ± 5.72abc	22.92 ± 4.98abc	17.23 ± 4.86bc	0.00 ± 0.00c	*p* < 0.001

* Manufacturer recommended dose. ^a^ Index of fecundity (IF) = mass of eggs laid/mass of engorged females. ^b^ %IO = [(IF control − IF treated)/(IF control) × 100]. SE: standard error. Mean IF/%IO followed by same letters were not statistically significant (*p* > 0.05).

**Table 4:** Lethal concentration (LC_50%_) values and resistance ratios (RRs) for amitraz and deltamethrin obtained using adult immersion test (AIT) on field populations of *R. (B.) microplus* from communal farms.

Agro-Climatic Region	Communal Farm/Dip Tank	Slope ± SE	R^2^	LC_50%_(ppm)	CI 95% LC_50%_ (ppm)	^b^ RR
**Amitraz**						
Inland	Mpafane	1.04 ± 0.20	0.89	46.26	32.16–66.48	0.28
	Mveso	1.40 ± 0.23	0.91	75.24	58.92–96.08	0.46
	Tyalarha	1.63 ± 0.23	0.92	530.2	436.9–643.4	3.21 ^c^
Coastal	Mapuzi	1.72 ± 0.22	0.94	378.9	318.9–450.1	2.30 ^c^
	Zanci	1.05 ± 0.28	0.71	143.6	83.81–245.9	0.87
	Bozisa	1.83 ± 0.36	0.88	438.0	339.5–565.2	2.65 ^c^
	^a^ IVRI-I line	3.49 ± 0.46	0.94	165.0	155.7–174.9	-
**Deltamethrin**						
Inland	Mpafane	2.44 ± 0.51	0.87	195.5	161.5–236.7	14.59 ^c^
	Baziya	1.06 ± 0.14	0.90	95.76	74.41–123.2	7.15 ^c^
	Tyalarha	0.60 ± 0.12	0.70	11.29	5.85–21.76	0.84
Coastal	Mapuzi	1.09 ± 0.21	0.79	62.53	42.09–92.90	4.67 ^c^
	Ndakana	1.06 ± 0.18	0.88	54.94	41.09–73.45	4.10 ^c^
	Zanci	1.64 ± 0.25	0.92	56.38	45.32–70.14	4.21 ^c^
	^a^ IVRI-I line	4.51 ± 0.28	0.99	13.40	12.40–14.50	-

^a^ The susceptible reference *R. (B.) microplus* IVRI-I line is maintained at the Entomology Laboratory of the Indian Veterinary Research Institute and is used as a standard for the assessment of susceptibility or resistance against various acaricides [31]. ^b^ Resistance ratio (RR): LC_50%_ of field isolate/LC_50%_ of susceptible strain. ^c^ Level of resistance is significant. LC: lethal concentration; CI: confidence intervals.

### 3.3. Amitraz and Deltamethrin Resistance Status from Field-Derived R. (B.) microplus Populations Using the Larval Immersion Test (LIT)

In a number of LIT bioassays that were conducted in this study, control mortality data obtained were often greater than 10%, and based on recommendations from FAO [16], the entire results had to be discarded. Therefore, due to the limited availability of larvae, reliable concentration-dependent mortality data for assessment of amitraz and deltametrin resistance were only obtained from four field tick populations of *R. (B.) microplus* (Table 5). The tick populations assessed showed LC_50%_ values ranging between 14 and 539 ppm and 3 and 9 ppm for amitraz and deltamethrin, respectively (Table 5). Low mortality slope values (<1.2) of the regression and LC_50%_ values with wide 95% confidence intervals obtained in three of the tick populations assessed for amitraz and deltamethrin resistance indicate heterogeneity in response to increasing concentrations of the acaricides. The coefficients of determination of all the estimations were greater than 74%, indicating good fitting of the data in the Probit model. Furthermore, all the tick populations assessed showed evidence of resistance (mean percentage of larval mortality < 90% at the DD) to deltamethrin (Table 6). However, only one (Mpafane) tick population was considered susceptible to amitraz (mean percentage of larval mortality > 90% at the DD). At 4XDD, all field tick populations were susceptible to deltamethrin, and at 8XDD, the tick populations were susceptible to both amitraz and deltamethrin. Statistically (*p* < 0.05) significant differences were noted between the mean percentage larval mortality values of the DD and control treatments for both acaricide chemical products. 

## 4. Discussion

In the Eastern Cape Province (ECP) of South Africa, both *Rhipicephalus (Boophilus) decoloartus and R. (B.) microplus* occur together in some areas [2]. The data on tick identification confirm the displacement of the indigenous *R.* (*B.*) *decoloratus* by *R. (B.) microplus* in the eastern region of the ECP [2], where this study was conducted. In communal areas of ECP, it is mandatory for resource-poor farmers to use plunge dipping for the control of ticks and TBDs on cattle [33]. However, veterinarians and cattle farmers have often complained of tick-acaricide treatment failures on communal farms of the ECP [24]. The present data provide the rate of amitraz and deltamethrin resistance development in *R. (B.) microplus* populations from selected communal farms with reported cases of tick-acaricide treatment failure in the King Sabata-Dalindyebo Municipality (KSDM). 

The adult immersion test (AIT) [16] and larval immersion test (LIT) [17] have been used to test for resistance to acaricides, where resistance mechanisms are unknown. Data obtained when AIT and LIT are used to assess resistance cannot be directly compared, as the former assesses acaricide resistance on engorged females, while the latter is conducted on tick larvae [34]. Mekonnen et al. [34] found that both AIT and AIT detected resistance to amitraz and cypermethrin in *R. (B.) decoloratus* on dairy farms of the ECP, albeit the results obtained with AIT often differed from those with LIT. In other studies, Mekonnen et al. [20] and Yawa et al. [21] detected cypermethrin and amitraz-resistant populations of *R. (B) decoloratus* with the LIT. The current study observed that both bioassays detected higher frequencies (>50%) of resistance development in field *R. (B.) microplus* populations to deltamethrin and amitraz. In their study, Ntondini et al. [9] found *R. (B) microplus* ticks on communally grazed cattle of the ECP exhibiting lower rates of acaricide resistance development to amitraz and cypermethrin. However, it is expected that, with incessant and indiscriminate use of acaricide chemicals over the last 12 years, the number of field *R. (B.) microplus* populations with acaricide resistance development should be on the rise. Initially, within a tick population, there is always a low rate of increase in the number of resistant individuals. Nevertheless, over time and as a result of continuous selection pressure from the application of acaricides, the frequency of resistant individuals in a population becomes higher [11]. In South Africa, it was shown that 18 months of cattle dipping resulted in the emergence of acaricide resistance by *R. (B.) decoloratus* to synthetic pyrethroids [35]. According to Jonsson et al. [36], field tick populations have a higher chance of developing resistance when acaricides are applied more than 5 times/year. In communal farming areas of the ECP, treatment of cattle against ticks is done at a frequency of 24–48 times/year during the summer season [24]. Other tick control malpractices that have favored the increase in selection pressure on ticks over time and led to the establishment of resistance in *R. (B.) microplus* field populations in communal farms of the ECP include the absence of acaricide rotation, poor surveillance of acaricide resistance, lack of training on the judicious use of acaricides, indiscriminate use of commercially acquired or adulterated acaricides, and use of acaricides at high frequencies and concentrations [9,20,21,24,34,37]. 

Amitraz and synthetic pyrethroid (SP) chemical compounds, including cypermethrin and deltamethrin, have been extensively used for the control of ticks on cattle at communal dip tanks of the Eastern Cape Province [21]. Synthetic pyrethroids are the most frequently used veterinary product in the ECP of South Africa [38], and amitraz has been used extensively for the control of ticks in South Africa [14]. The use of amitraz became popular when SP resistance problems began to hinder tick control efforts [11]. However, its use on communal farms of the ECP has been suspended, owing to frequent farmer reports of its failure to control ticks on cattle [24]. Amitraz-resistant field *Riphicephalus (Boophilus)* spp. populations have been reported from both commercial [20] and communal [9,20,21] farms of the ECP. There are also several reports of field tick resistance against amitraz from different parts of the globe [36,39,40]. During the period of the study, deltamethrin was actively in use at all the communal dip tanks sampled. The use of deltamethrin was reintroduced by the government as an acaricide rotation strategy when amitraz resistance problems were frequently reported [24]. Deltamethrin has been in continuous use at the communal dip tanks of the KSDM for a period of more than five years [24]. In addition, other easily accessible commercial SP compounds such as Deadline^®^ (flumethrin 1%) and Maxipour^®^ (flumethrin 1%) pour-ons have been used by cattle farmers to supplement the state-funded dipping program [10,24]. The prolonged and indiscriminate use of SP compounds at these communal farms has probably contributed to the selection of deltamethrin-resistant *R. (B.) microplus* field populations [13,20]. Cypermethrin-resistant field *Riphicephalus (Boophilus*) spp. populations have also been reported from both commercial [20] and communal [9,20,21] farms of the ECP. The development of resistance by field tick populations to cypermethrin on communal farms in the ECP might have eventually led to the observed exhibition of side resistance to deltamethrin [13,34].

The Drummond et al. [18] adult immersion test (AIT) with a number of modifications has been adopted in many countries, including Benin [41], Brazil [42], India [43,44,45,46,47], and Australia [48], for the diagnosis of resistance of *R. (B.) microplus* against SP and amitraz. However, due to its limitation of the unavailability of sufficient undamaged, suitable (size > 4 mm), and fully engorged female ticks for the bioassay [16], suitable specimens could not be obtained from all the sampled communal farms for assessment of resistance to both acaricide compounds. Additionally, data from the Indian susceptible reference *R. (B.) microplus* line were used in the quantification of resistance ratios (RRs) of ticks to amitraz and deltametrin as a consequence of limited data on regional or country-specific reference *R. (B.) microplus* lines. The development of resistance in tick populations is usually affected by regional or country-specific factors such as geographical location, socio-economic status of farmers, breed of cattle, dose, and acaricide treatment frequency [49]. The Shaw [17] larval immersion test (LIT) is not so widely used in the detection of acaricide resistance and has not been promoted by FAO [13]. However, the LIT is reported to have been used successfully in South Africa for detecting resistance in cypermethrin and amitraz [9,20,34]. Additionally, in South Africa, the LIT is reported to have been the preferred bioassay technique in the National Survey of Acaricide Resistance [9]. Although quantification of resistance ratios (RRs) with LIT was omitted, the low mortality slope values (<1.2) of the regression and LC_50%_ values with wider 95% confidence intervals in a majority of the field tick populations assessed with amitraz and deltametrin using the LIT is indicative of the presence of a heterogeneous response in ticks to increasing concentrations of the acaricides [50]. This is a common characteristic of resistant field tick populations with high genetic variations, exhibiting intermediate levels of resistance to acaricides [13,50]. Most researchers opine that lower slopes of concentration–mortality lines with corresponding wider 95% confidence intervals in LC_50%_ and LC_90%_ from acaricide bioassays indicate tick populations that are resistant to acaricides [49,51]. 

## 5. Conclusions

Field populations of *R. (B.) microplus* ticks from communal farms of the ECP of South Africa have developed acaricide resistance to amitraz and deltametrin. In order to mitigate the development of tick resistance to these acaricides, a practical tick control strategy is necessary. This strategy should include farmer education on tick control malpractices and regular monitoring of acaricide resistance on farms where tick-acaricide failure is reported by farmers. Other strategies consist of using mixtures of acaricides with different modes of action or acaricide rotation practice. Hence, resistance profiles of other classes of acaricides used by farmers to supplement the state dipping program and that were not assessed in this study should be investigated, so as to provide a full range of options for acaricide rotation. In addition, an investigation into the underlying molecular mechanisms involved in *R. (B.) microplus* resistance to the acaricides assessed in this study is recommended. 

## Figures and Tables

**Table 5:** Lethal concentration (LC_50%_) estimates for amitraz and deltamethrin obtained using the larval immersion test (LIT) on field populations of *R. (B.) microplus* from communal farms.

Communal Farm/Dip Tank	Slope ± SE	R^2^	LC_50%_ (ppm)^3^	CI 95% LC_50%_ (ppm)
**Amitraz**				
**Bozisa**	0.89 ± 0.21	0.75	117.4	69.54–198.2
**Mpafane**	0.90 ± 0.14	0.92	14.05	8.34–23.66
**Tyalarha**	0.64 ± 0.12	0.78	33.34	16.31–68.15
**Mapuzi**	1.34 ± 0.19	0.89	538.3	423.1–685.0
**Deltamethrin**				
**Ndakana**	1.18 ± 0.12	0.95	8.74	7.33–10.43
**Mpafane**	3.44 ± 0.26	0.97	3.28	2.21–4.86
**Baziya**	1.10 ± 0.18	0.88	5.99	4.21–8.54
**Mapuzi**	1.13 ± 0.28	0.79	8.23	5.14–13.18

LC: lethal concentration; CI: confidence intervals.

**Table 6:** Mean percentage larval mortality (%MLM) for amitraz and deltamethrin, obtained using the larval immersion test (LIT) on field populations of *R.* (*B*.) *microplus* from communal farms.

Communal Farm Area	Total Number of Larvae	Mean Percentage Larval Mortality (%MLM ± SE)								
		Amitraz Concentration (ppm)								
		2000	1000	500	250 *	125	62.5	31.25	Control	*p*-Value
Mpafane Tyalarha Mapuzi	2312	100 ± 0.00a	92.56 ± 3.57ab	91.53 ± 4.20ab	90.06 ± 0.78ab	87.90 ± 3.86ab	88.56 ± 2.69ab	78.88 ± 6.66b	0.00 ± 0.00c	*p* < 0.001
	1912	97.23 ± 1.39a	87.69 ± 2.85ab	82.42 ± 5.60ab	79.17 ± 8.28ab	70.22 ± 5.74b	69.36 ± 3.81b	69.42 ± 5.89b	0.00 ± 0.00c	*p* < 0.001
	2476	98.83 ± 0.69a	84.30 ± 4.80b	72.60 ± 4.42bc	62.07 ± 1.27c	46.30 ± 0.84d	40.33 ± 1.76d	38.73 ± 1.18d	0.00 ± 0.00e	*p* < 0.001
Bozisa	2136	94.18 ± 7.41a	86.35 ± 12.93a	84.13 ± 9.32a	65.77 ± 5.37b	57.76 ± 11.04bc	47.96 ± 10.06bc	46.10 ± 5.92b	0.00 ± 0.00c	*p* < 0.001
**Deltamethrin Concentration (ppm)**										
		**400**	**200**	**100**	50 *	**25**	**12.5**	**6.25**	**Control**	***p*-Value**
Mpafane Baziya Mapuzi	1876	94.79 ± 0.40a	92.62 ± 3.79ab	90.90 ± 2.78ab	86.90 ± 2.27ab	82.83 ± 0.06ab	76.89 ± 1.93b	75.51 ± 1.42b	0.00 ± 0.00c	*p* < 0.001
	2027	98.01 ± 4.01a	93.39 ± 1.85ab	86.65 ± 5.34ab	73.71 ± 8.38b	72.07 ± 4.16b	70.35 ± 3.92b	54.68 ± 8.11b	0.00 ± 0.00c	*p* < 0.001
	2189	98.75 ± 7.60a	94.95 ± 7.75ab	89.02 ± 1.34ab	87.21 ± 6.78ab	82.82 ± 4.38b	67.69 ± 6.92bc	61.33 ± 10.08c	0.00 ± 0.00d	*p* < 0.001
Ndakana	2372	100 ± 0.00a	97.29 ± 3.54a	87.20 ± 3.83ab	76.59 ± 1.47b	72.28 ± 2.69b	67.82 ± 0.82b	62.23 ± 1.66b	0.00 ± 0.00c	*p* < 0.001

* Manufacturer recommended dose. SE: standard error; %MLM with the same small case letters are not significantly different.

## Data Availability

The data presented in this study are available on request from the corresponding author. The data are not publicly available as they contain information that could compromise the privacy of the research participants.

## Supplementary Materials

The following are available online at https://www.mdpi.com/article/10.3390/pathogens12070875/s1. Figure S1: morphological keys used in the identification of *Rhipicephalus Boophilus* species, Walker et al. (2003) [25]. Figure S2: BLASTn results showing the alignment of the *Rhipicephalus microplus* of this study and a corresponding sequence.

## List of Abbreviations

KSDM, King Sabata Dalindyebo municipality; ECP, Eastern Cape Province; AIT, adult immersion tests; LIT, larval immersion tests; ppm, parts per million; SP, synthetic pyrethroids; AHTs, animal health technicians; CAHWs, community animal health workers; PCR, polymerase chain reaction; DD, diagnostic dose; LC, lethal concentration.
