# Supplementary material for: Acaricide Resistance Development in Rhipicephalus (Boophilus) microplus (Acari: Ixodidae) Populations against Amitraz and Deltamethrin on Communal Farms of the King Sabata Dalindyebo Municipality, South Africa
Source: Pathogens. 2023 Jun 26;12(7):875. doi: 10.3390/pathogens12070875 (PMC10383965; doi:10.3390/pathogens12070875)
Supplement: Supplementary file 1 [file pathogens-12-00875-s001.zip › pathogens-2433778-supplementary.pdf]

Acaricide Resistance Development in *Rhipicephalus (Boophilus) microplus* (Acari: Ixodidae) Populations against Amitraz and Deltamethrin on Communal Farms of the King Sabata Dalindyebo Municipality, South Africa

William Diymba Dzemo [1,2,*], Patrick Vudriko [3], Tsepo Ramatla [2] and Oriel Thekisoe [2]

[1] Department of Biological and Environmental Sciences, Faculty of Natural Sciences, Walter Sisulu University, Mthatha 5117, South Africa
[2] Unit for Environmental Sciences and Management, North-West University, Potchefstroom 2531, South Africa; ra21205450@gmail.com (T.R.); oriel.thekisoe@nwu.ac.za (O.T.)
[3] Research Center for Tropical Diseases and Vector Control, Department of Veterinary Pharmacy, Clinics and Comparative Medicine, College of Veterinary Medicine, Animal Resources and Biosecurity, Makerere University, Kampala, P.O Box 7062, Uganda; patrick.vudriko@mak.ac.za
* Correspondence: wdzemo@wsu.ac.za

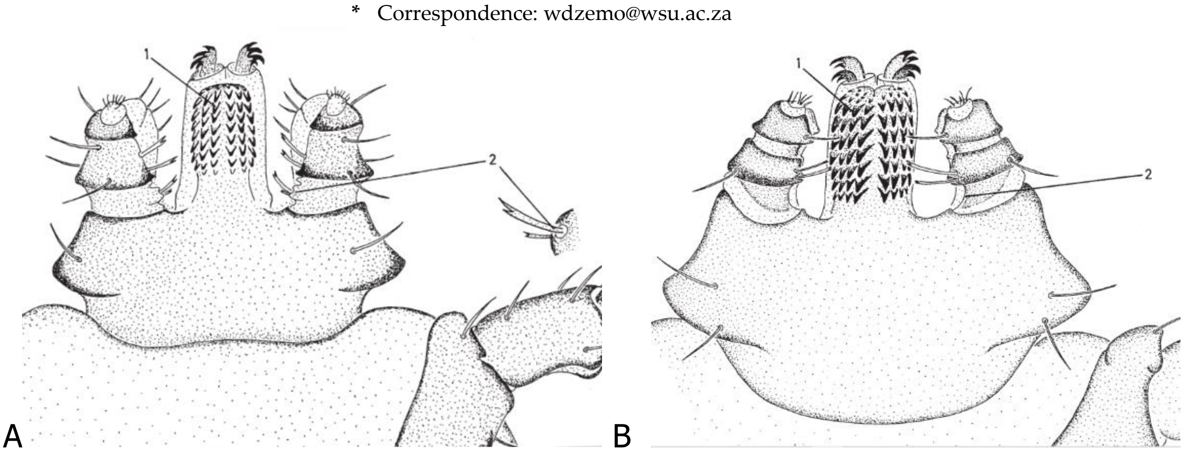

Supplementary Figure S1: Morphological keys used in the identification of *Rhipicephalus Boophilus* species, Walker et al. (2003). A: *Rhipicephalus B. microplus*; B: *Rhipicephalus B. decoloratus*.

Sequences producing significant alignments Download Select columns Show 100

select all *100 sequences selected* GenBank Graphics Distance tree of results MSA Viewer

Description	Scientific Name	Max Score	Total Score	Query Cover	E value	Per. Ident	Acc. Len	Accession
Rhipicephalus microplus isolate HAI2 internal transcribed spacer 2, partial sequence	Rhipicephalus mi...	1995	2109	98%	0.0	98.17%	1148	MK224566.1
Rhipicephalus microplus isolate HE1 internal transcribed spacer 2, partial sequence	Rhipicephalus mi...	1995	2109	98%	0.0	98.17%	1151	MK224560.1
Rhipicephalus microplus isolate JX-2 voucher NCCDCITS171 5.8S ribosomal RNA gene, partial sequence; intern...	Rhipicephalus mi...	1995	2109	98%	0.0	98.17%	1342	MG721035.1
Rhipicephalus microplus clone 60_8908_763 18S ribosomal RNA gene, internal transcribed spacer 1, 5.8S riboso...	Rhipicephalus mi...	1995	2109	98%	0.0	98.17%	8908	KY457506.1
Rhipicephalus microplus isolate 11-2 5.8S ribosomal RNA gene, partial sequence; internal transcribed spacer 2, c...	Rhipicephalus mi...	1995	2109	98%	0.0	98.17%	1521	KX450287.1
Rhipicephalus microplus isolate BomiB 5.8S ribosomal RNA gene, partial sequence; internal transcribed spacer 2...	Rhipicephalus mi...	1995	2109	98%	0.0	98.17%	1287	KC503273.1
Rhipicephalus microplus isolate 2Ken 5.8S ribosomal RNA gene, partial sequence; internal transcribed spacer 2,...	Rhipicephalus mi...	1995	2109	98%	0.0	98.17%	1285	KC503264.1
Rhipicephalus microplus isolate KN159 5.8S ribosomal RNA gene, partial sequence; internal transcribed spacer 2...	Rhipicephalus mi...	1995	2109	98%	0.0	98.17%	1324	MW227651.1
Rhipicephalus microplus isolate 8023 5.8S ribosomal RNA gene, partial sequence; internal transcribed spacer 2,...	Rhipicephalus mi...	1995	2109	98%	0.0	98.17%	1346	JQ625709.1
Rhipicephalus microplus isolate 9023 5.8S ribosomal RNA gene, partial sequence; internal transcribed spacer 2,...	Rhipicephalus mi...	1995	2109	98%	0.0	98.17%	1345	JQ625705.1
Rhipicephalus microplus 5.8S ribosomal RNA gene, partial sequence; internal transcribed spacer 2, complete seq...	Rhipicephalus mi...	1995	2109	98%	0.0	98.17%	1340	JF758642.1
Rhipicephalus microplus 5.8S ribosomal RNA gene, partial sequence; internal transcribed spacer 2, complete seq...	Rhipicephalus mi...	1995	2109	98%	0.0	98.17%	1342	JF758640.1
Rhipicephalus microplus isolate HE3 internal transcribed spacer 2, partial sequence	Rhipicephalus mi...	1991	2105	98%	0.0	98.08%	1151	MK224562.1
Rhipicephalus microplus isolate LD1 internal transcribed spacer 2, partial sequence	Rhipicephalus mi...	1989	2103	98%	0.0	98.08%	1151	MK224573.1
Rhipicephalus microplus isolate HE4 internal transcribed spacer 2, partial sequence	Rhipicephalus mi...	1989	2103	98%	0.0	98.08%	1151	MK224563.1
Rhipicephalus microplus isolate GZ1 internal transcribed spacer 2, partial sequence	Rhipicephalus mi...	1989	2103	98%	0.0	98.08%	1151	MK224551.1
Rhipicephalus microplus isolate Itanagar 5.8S ribosomal RNA gene, partial sequence; internal transcribed spacer ...	Rhipicephalus mi...	1989	2103	98%	0.0	98.08%	1341	MK625221.1
Rhipicephalus microplus isolate Aizawl 5.8S ribosomal RNA gene, partial sequence; internal transcribed spacer 2...	Rhipicephalus mi...	1989	2103	98%	0.0	98.08%	1477	MK621182.1

Supplementary Figure S2: BLASTn results showing the alignment of the *Rhipicephalus microplus* of this study and a corresponding sequence.
